# DNA Damage Activates the SAC in an ATM/ATR-Dependent Manner, Independently of the Kinetochore

**DOI:** 10.1371/journal.pgen.1000015

**Published:** 2008-02-29

**Authors:** Eun Mi Kim, Daniel J. Burke

**Affiliations:** Department of Biochemistry and Molecular Genetics, University of Virginia Medical Center, Charlottesville, Virginia, United States of America; National Institute of Diabetes & Digestive & Kidney Diseases, United States of America

## Abstract

The DNA damage checkpoint and the spindle assembly checkpoint (SAC) are two important regulatory mechanisms that respond to different lesions. The DNA damage checkpoint detects DNA damage, initiates protein kinase cascades, and inhibits the cell cycle. The SAC relies on kinetochore-dependent assembly of protein complexes to inhibit mitosis when chromosomes are detached from the spindle. The two checkpoints are thought to function independently. Here we show that yeast cells lacking the DNA damage checkpoint arrest prior to anaphase in response to low doses of the DNA damaging agent methyl methane sulfonate (MMS). The arrest requires the SAC proteins Mad1, Mad2, Mad3, Bub1, and Bub3 and works through Cdc20 and Pds1 but unlike the normal SAC, does not require a functional kinetochore. Mec1 (ATR) and Tel1 (ATM) are also required, independently of Chk1 and Rad53, suggesting that Mec1 and Tel1 inhibit anaphase in response to DNA damage by utilizing SAC proteins. Our results demonstrate cross-talk between the two checkpoints and suggest that assembling inhibitory complexes of SAC proteins at unattached kinetochores is not obligatory for their inhibitory activity. Furthermore, our results suggest that there are novel, important targets of ATM and ATR for cell cycle regulation.

## Author Summary

Genome integrity is assured, in part, by regulatory systems called “checkpoints” that assure that cells do not improperly progress through the cell cycle. The DNA damage checkpoint assesses the status of DNA replication and inhibits cell cycle progression when the cell makes mistakes in DNA replication or when the cell has been assaulted by a DNA damaging agent from the environment. The checkpoint allows the cell time to repair the DNA and then permits the cell cycle to resume. There is a separate “spindle checkpoint” that monitors whether chromosomes are properly attached to the spindle and if so, allows cells to proceed through mitosis. The DNA damage checkpoint and the spindle checkpoint assure that daughter cells receive the correct number of chromosomes that are identical in DNA sequence. Here we show that the two checkpoints are not independent but that they cooperate to restrict mitotic progression in the face of DNA damage. We show that the spindle checkpoint can be induced by DNA damage and that there is a novel kinetochore independent mechanism to activate the spindle checkpoint proteins. In addition, we implicate the ATM and ATR kinases as kinetochore-independent activators of the spindle checkpoint.

## Introduction

Two evolutionarily conserved checkpoints, the DNA damage checkpoint and the spindle assembly checkpoint (SAC), control the fidelity of chromosome segregation. The DNA damage checkpoint responds to a variety of DNA lesions and controls entry into S phase, completion of S phase and entry into mitosis [1],[2]. The DNA damage checkpoint is a signal transduction network consisting of sensors, signal transducers and downstream effectors. Central to the signal transduction network in budding yeast are two phosphotidylinositol 3’ kinase-like kinases (PIKKs), Mec1 (the yeast homolog of ATM and Rad3-related protein, abbreviated ATR) and Tel1 (the yeast homolog of the ataxia-telangiectasia-mutated protein abbreviated ATM) [1],[3],[4]. Mec1 and Tel1 activate the protein kinase transducers Rad53, Chk1 and Dun1 leading to cell cycle arrest and induction of DNA repair genes [5]–[9].

The SAC responds to chromosomes that are either unattached from the spindle or are not under tension and delays the metaphase to anaphase transition [10]. The kinetochore has an integral role in the SAC and a popular model is that the kinetochore initiates checkpoint signaling by being the site of assembly of inhibitory complexes of SAC proteins that inhibit mitosis [10],[11]. The inhibitory complexes are made up of combinations of the evolutionarily conserved proteins Bub1 Bub3, Mad1, Mad2, and Mad3 (BubR1 in higher cells) but the exact details of their assembly and inhibitory activities are unknown [12]–[15]. The two checkpoints share a common target to regulate mitosis. Pds1 (securin in higher organisms) is an anaphase inhibitor that is stabilized by two different mechanisms when the two checkpoints are activated. Pds1 is phosphorylated and thereby stabilized by the DNA damage checkpoint [16]. The SAC stabilizes Pds1 by inhibiting Cdc20, the specificity factor for an E3-ubiquitin ligase called the anaphase-promoting complex (APC) that is responsible for the proteolysis of Pds1 [17],[18].

There are indications, from yeast to humans, that the DNA damage checkpoint and the SAC have overlapping functions. Laser microbeam-induced DNA damage during late prophase in some human cell lines delays progress through metaphase in a P53-independent manner and the delay is abrogated by inhibiting Mad2 [19]. Cells derived from a mouse mutant, heterozygous for a deletion of BubR1, are defective in the response to genotoxic agents suggesting that BubR1 is limiting in the DNA damage response [20]. *Drosophila* grapes mutants (*grp*), lacking the homolog of Chk1, delay anaphase after X-irradiation and the delay is dependent on BubR1 [21]. Camptothecin induces a mitotic delay in fission yeast cells lacking the DNA damage checkpoint and the delay requires Mad2 [22]. In addition, fission yeast Mad2 plays a minor role in the mitotic delay imposed by growing cells in the presence of the ribonucleotide reductase inhibitor hydroxyurea (HU) but Mad1, Bub1 and Mad3 do not play a role [23]. Budding yeast cells lacking the DNA damage checkpoint (*rad9 rad24* double mutants) and compromised for DNA replication by mutations in *cdc2-1, pol1-17, mcm2-1,*or *mcm3-1* delay in mitosis in a Mad2-dependent fashion [24]. Compromising both DNA replication and the DNA damage checkpoint in *orc1-161 rad53-11* cells causes a delay in mitosis in a Mad2 and Bub1-dependent manner [25]. The DNA alkylating agent, methyl methane sulfonate (MMS), HU, and ultraviolet light also induces a mitotic delays in cells lacking the DNA damage checkpoint and the delays require Mad1 and Mad2 [24],[26]. Models to explain why such diverse mutants and treatments cause a SAC-dependent mitotic delay propose that kinetochores may be damaged or poorly assembled due to aberrant centromere DNA replication or defects in sister chromatid cohesion may result in a loss of tension across sister kinetochores [23]–[27]. These models are in accord with the proposition that the SAC signal is generated at kinetochores that are either detached from the mitotic spindle or from kinetochores that are on chromatids lacking tension, as would be caused by defective cohesion [10], [11], [28]–[31]. However, explanations invoking a role for the kinetochore in a DNA damage response are harder to reconcile with observations that double strand DNA breaks near telomeres in yKu70Δ cells or a single double strand break induced by *HO* at *URA3* induces a mitotic delay in cells lacking the DNA damage checkpoint [32],[33]. It was proposed that telomere proximal double strand breaks in cells lacking Yku70 results in dicentric chromosomes that are known to activate the SAC, presumably by altering tension at kinetochores [32]. The single double strand break introduced at *URA3* causes a delay in the second cell cycle after *HO* induction which may also reflect the formation of dicentric chromosomes as the source of the SAC signal [33].

In this study we test the model that the kinetochore is required to activate the SAC proteins in response to DNA damage. We show that cells arrest prior to anaphase when grown in the presence of MMS and that the arrest requires the SAC proteins Mad1, Mad2, Mad3, Bub1 and Bub3. Surprisingly, temperature-sensitive *ndc10-1* cells that are devoid of kinetochores also arrest in response to MMS suggesting that the kinetochore is not required to convert the SAC proteins into inhibitors under these conditions. We show that the downstream effectors of the SAC (Cdc20 and Pds1) are required for the arrest suggesting that the inhibition by the checkpoint proteins works through the canonical SAC. Furthermore, we show that the SAC is capable of restraining anaphase in response to MMS in cells lacking the DNA damage checkpoint and that the yeast homologs of ATM (Tel1) and ATR (Mec1) are required for the SAC-dependent arrest suggesting that the PIKKs are required to activate both the DNA damage checkpoint and the SAC. These studies reveal an intimate relationship between the DNA damage and SAC pathways and highlight the importance of preventing anaphase in cells with damaged chromosomes.

## Results/Discussion

**Figure 1:**
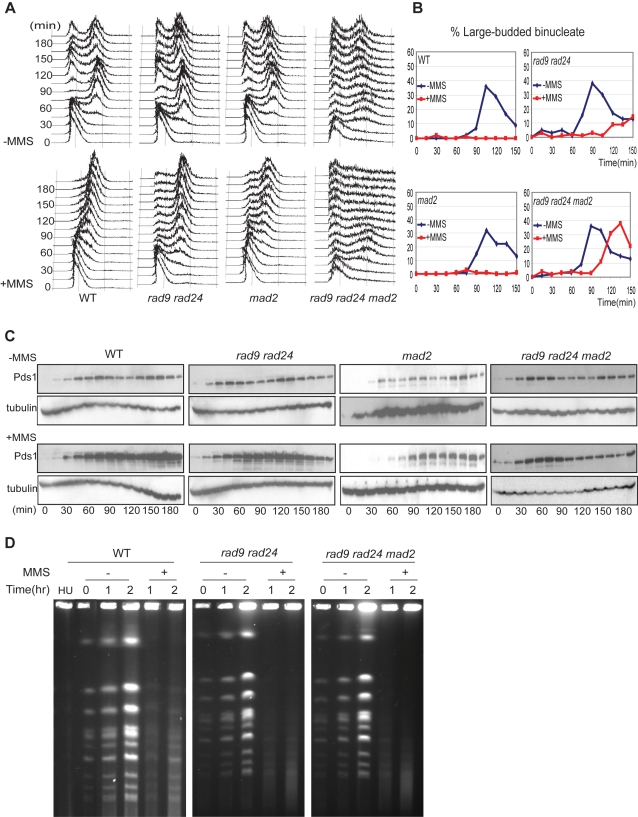
Mad2 is required to delay of mitosis in response to MMS. (A) Flow cytometry of wild type (WT) and mutant cells with the indicated genotypes that were arrested with α-factor and released into YPD medium in the presence or absence of 0.01% MMS. Cells were assayed every fifteen minutes. The *rad9 rad24* and *mad2* strains were tested twice, WT was tested six times, and *rad9 rad24 mad2* was tested three times. (B) The percentage of large budded bi-nucleate cells (anaphase) from panel A for wild type and indicated mutant cells after release into medium with or without MMS. At least 100 cells were counted for each time point. Cell morphologies indicative of other phases of the cell cycle are in Figure S2. (C) Pds1-13 Myc stability of wild type and mutants cells. Endogenous Pds1 was tagged with 13 copies of the Myc epitope. Protein extracts from the cells in (A) were prepared and Western blot analysis was performed with 9E10 mouse anti-Myc monoclonal antibody. Upper half was in the absence of MMS and lower half was in the presence of MMS. Western blots with anti-Tub2 (tubulin) were for loading control. (D) Wild type and cells of the indicated genotypes were arrested with α-factor and released into YPD medium with or without 0.01% MMS. HU indicates wild type cells arrested with 0.1 M hydroxyurea. Samples were taken every hour and chromosomes were separated by CHEF.

We applied several different assays to measure the mitotic delay in cells treated with MMS. Cells were arrested in G1 by growth in the presence of α-factor and then released to the cell cycle in the presence and absence of 0.01% MMS [24]. We monitored cell cycle progression by a combination of flow cytometry, cell morphology and Pds1 (securin) stability. Cells from four isogenic strains cycled normally in the absence of MMS as judged by DNA flow cytometry (Figure 1A, upper panels), cellular morphology (Figure 1B) and Pds1 stability (Figure 1C). MMS treated wild type and *mad2* cells delayed progress though S phase, as determined by flow cytometry and arrested with a G2/M content of DNA (Figure 1A, lower panels), prior to anaphase (Figure 1B) with high levels of Pds1 (Figure 1C) due to activation of the DNA damage checkpoint. *rad9 rad24* cells, lacking the DNA damage checkpoint, also delayed with a G2/M content of DNA when grown in the presence of MMS (Figure 1A, lower panel), failed to complete anaphase and accumulated as large budded cells with a single undivided nucleus (Figure 1B and Figure S2) and stabilized Pds1 (Figure 1C). The MMS-dependent mitotic delay was abrogated in *rad9 rad24 mad2* cells that failed to accumulate with a G2/M content of DNA (Figure 1A, lower panel), progressed into anaphase (Figure 1B and Figure S2) and failed to stabilize Pds1 (Figure 1C). We measured reproducibility of the response by analysis of multiple flow cytometry profiles (Figure S1A–S1D). Each experiment was performed between 2–6 times and duplicates for each of the flow cytometry experiments are shown including the mean percentage of cells with the G2/M content of DNA determined from the flow cytometry profiles along with the variance in those data. The range of measurements is shown for experiments performed twice and the standard deviation was calculated and is indicated as error bars at each time point for experiments done more than twice. These data confirm that MMS treatment of *rad9 rad24* cells lacking the DNA damage checkpoint cause a pre-anaphase delay that is dependent on Mad2 [24].

Haploid *rad9 rad24* cells delayed with a G2/M content of DNA suggesting that they had arrested after S phase. We used Clamped Homogeneous Electric Field (CHEF) gels to analyze whole chromosomes in cells treated with MMS to determine if the synchronized cells completed DNA replication in response to MMS treatment. CHEF gels are used to separate large (yeast chromosome-sized) fragments of DNA by electrophoresis and are useful for karyotyping yeast cells [34]. In addition, they can be used to determine if DNA replication is complete as chromosomes from cells with unreplicated DNA either do not enter the gel and therefore bands are not present or the DNA appears as faintly staining bands with smeared appearances [35]–[37]. Untreated wild type, *rad9 rad24* and *rad9 rad24 mad2* cells had normal CHEF karyotypes with clearly identified chromosomes (Figure 1D). Wild type cells treated with the ribonucleotide reductase inhibitor hydroxyurea (HU) do not complete DNA replication and chromosomes do not enter the gel and were not detected (Figure 1D). Chromosome staining in cells grown in the presence of MMS was weak in both *rad9 rad24* cells and *rad9 rad24 mad2* cells and was similar to wild type cells grown in the presence of HU (Figure 1D). We detected some chromosomal staining with a smeared appearance in wild type cells grown in the presence of MMS (Figure 1D). We conclude that cells grown under our conditions of 0.01% MMS and that delayed with a G2/M content of DNA had completed the bulk of DNA replication but accumulated with lesions, most likely stalled or collapsed replication forks.

**Figure 2:**
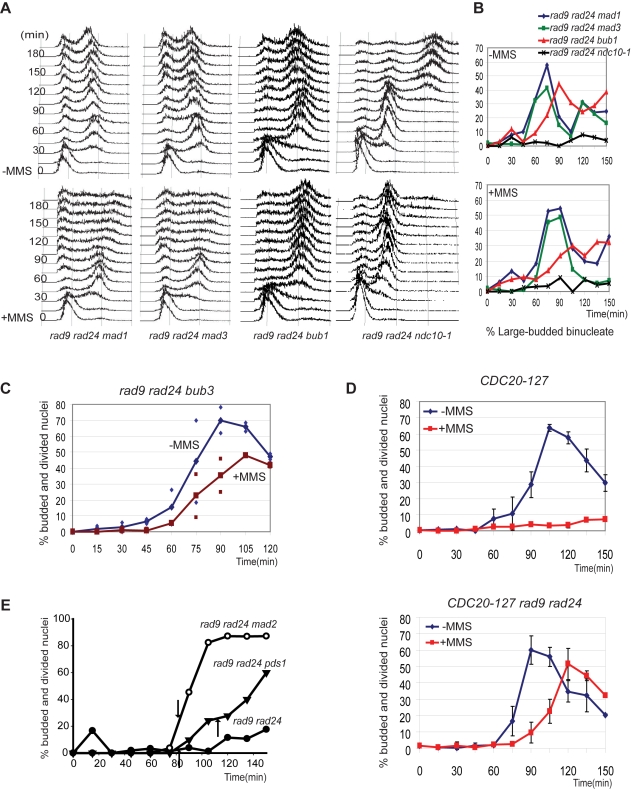
The SAC-dependent mitotic delay is independent of the kinetochore. (A) Flow cytometry of mutant cells with the indicated genotypes that were arrested with α-factor and released into YPD medium with or without 0.01% MMS. Cells were assayed every fifteen minutes. The *rad9 rad24 ndc10-1* cells were arrested with α-factor at 23°C for 3 hours, moved to 35°C for 1 hour to inactivate Ndc10, and then released to pre-warmed YPD at 35°C with or without MMS. All strains were tested twice except *rad9 rad24 bub1* which was tested 3 times. (B) The percentage of large budded bi-nucleate cells from panel A for wild type and indicated mutant cells after released into medium without MMS (upper panel) or with MMS (lower panel). Cell morphologies indicative of other phases of the cell cycle are in Figure S4. (C) The number of *rad9 rad24 bub3* cells that were budded with divided nuclei (anaphase) when grown in the presence or absence of MMS. Data from two independent experiments are represented. Solid lines are mean values and the dots are the independent measurements (range). (D) The SAC delay in response DNA damage requires APC^Cdc20^. The number of *CDC20-127* cells (upper panel) and *CDC20-127 rad9 rad24* cells (lower panel) that were budded with divided nuclei (anaphase) when grown in the presence or absence of MMS. Data from three independent experiments are represented. The means are plotted and standard deviation is indicated by error bars. Analyses of morphologies indicative of other phases of cell cycle are in Figure S5D. (E) The SAC delay in response DNA damage requires Pds1. Number of cells that were budded with divided nuclei (anaphase) when grown in the presence of MMS are graphed. Closed circles are *rad9 rad24* cells, open circles are *rad9 rad24 mad2* cells, and triangles are *rad9 rad24 pds1* cells. The arrows represent the time when 50% of the cells had completed anaphase when grown in the absence of MMS. Each point is the mean value of two independent experiments.

We assayed cell cycle progression in other SAC mutants to determine if all SAC proteins were required for the delay in response to MMS. Cells lacking the DNA damage checkpoint and either *mad1* or *mad3* proceeded normally through the cell cycle in the absence of MMS (Figure 2A, upper panels). The same cells did not accumulate with a G2/M content of DNA when grown in the presence of MMS (Figure 2A, lower panels) and reproducibility of the flow cytometry, as per Figure 1, is shown in Figure S3A and S3B. The *rad9 rad24 mad1* and *rad9 rad24 mad3* cells did not delay anaphase and completed nuclear division in the presence of MMS (Figure 2B and Figure S4). *bub1* cells delayed with a G2/M content of DNA in the presence and absence of MMS (Figure 2A). However, *bub1* cells failed to restrain anaphase and completed nuclear division slowly perhaps suggesting that they partially retain the delay (Figure 2A, 2B, and Figure S3C). Reproducibility for the flow cytometry of the *bub1* cells is shown in Figure S3C. It was difficult to determine the response of *rad9 rad24 bub3* cells by the same assay because of a high degree of inviability in the strain which made flow cytometry difficult to interpret. We assayed cell cycle progression by arresting cells in G1 with α-factor and allowed sufficient time for the viable cells to form mating projections. We released the cells and monitored the progression of only the cells with mating projections that subsequently budded and determined whether they completed nuclear division. Both treated and untreated cells completed nuclear division although MMS treated *bub3* cells slowly entered into anaphase (Figure 2C). We conclude that *bub3*, like *bub1*, abrogates the delay.

The kinetochore is required for the SAC and is thought to act as a platform that recruits checkpoint proteins when microtubules are unattached and assembles them into novel complexes that inhibit mitosis [10],[11]. Temperature sensitive *ndc10-1* cells are unable to assemble kinetochores and are unable to arrest in mitosis in response to nocodazole, a benzimidazole drug that depolymerizes microtubules [11],[38],[39]. Therefore *ndc10-1* cells lack the SAC at the restrictive temperature. We synchronized haploid *rad9 rad24 ndc10-1* cells with α-factor at 23°C, incubated the cells at 35°C for 1 hour to inactivate Ndc10 and then released the cells to allow them to progress through the cell cycle at the restrictive temperature. Chromosomes lacking kinetochores are unable to be segregated at mitosis and remain in the mother cell. DNA replication in the next cell cycle causes an increase in ploidy. *ndc10-1* cells, untreated with MMS, completed S phase and had a 2C content of DNA and then proceeded to the next cell cycle and increased the ploidy producing cells with a 4C content of DNA (Figure 2A, upper panel, reproducibility shown in Figure S3D). Wild type cells cycled normally in the absence of MMS at 35°C and did not produce cells with a 4C content of DNA (not shown). Therefore, the *ndc10-1* cells with a 4C content of DNA are the result of inactivating the kinetochore during the 1 hour incubation at 35°C. The same *ndc10-1* cells delayed in the first mitosis when grown in the presence of MMS (Figure 2A, lower panel and Figure S3D). Therefore kinetochores are not required for SAC-dependent inhibition of anaphase in response to MMS.

The SAC prevents the metaphase-to-anaphase transition by inhibiting the ubiquitylation and degradation of Pds1 by the APC. The target of the SAC is the APC regulatory subunit Cdc20 [18],. We determined if MMS inhibits anaphase through APC^Cdc20^ inhibition using *CDC20-127*; a dominant checkpoint-defective allele that produces a protein unable to bind Mad2 [40]. We generated *CDC20-127* (*CDC20^Y205N^*) by site directed mutagenesis, confirmed it by DNA sequencing and replaced the endogenous allele by a one-step gene replacement. *CDC20-127* and *CDC20-127 rad9 rad24* cells were delayed with a G2/M content of DNA in the absence of MMS (Figures S5A and S5B, upper panels) and cells completed nuclear division (Figure 2D). Reproducibility is shown in Supplementary Figure S5. *CDC20-127* cells delayed with a G2/M content of DNA when grown in the presence of MMS and delayed entry into anaphase (Figure S5A and Figure 2D, upper panel). In contrast, *CDC20-127 rad9 rad24* cells, grown in the presence of MMS, did not delay with a G2/M content of DNA, failed to restrain anaphase (Figure S5B and Figure 2D, lower panel) and did not stabilize Pds1 (Figure S5C). We conclude that *CDC20-127* abrogated the delay in response to MMS in *rad9 rad24* cells. Therefore, MMS induces a delay in *rad9 rad24* cells by promoting Mad2 binding to Cdc20 and inhibiting APC^Cdc20^.

A hypomorphic *top2-B44* mutant, with reduced activity of type 2 topisomerase, delays the onset of anaphase using SAC proteins independently of Pds1 suggesting a novel mitotic topoisomerase II checkpoint [42]. We assayed *pds1* cells using the assay described above for *bub3* cells to determine if *rad9 rad24* cells treated with MMS utilize this novel pathway. Growth in the presence of MMS delayed anaphase of *rad9 rad24* cells but not *rad9 rad24 mad2* and *rad9 rad24 pds1* cells (Figure 2E). Therefore the delay in response to MMS works through Cdc20 and Pds1 and is different from the one reported for partial topoisomerase inhibition.

**Figure 3:**
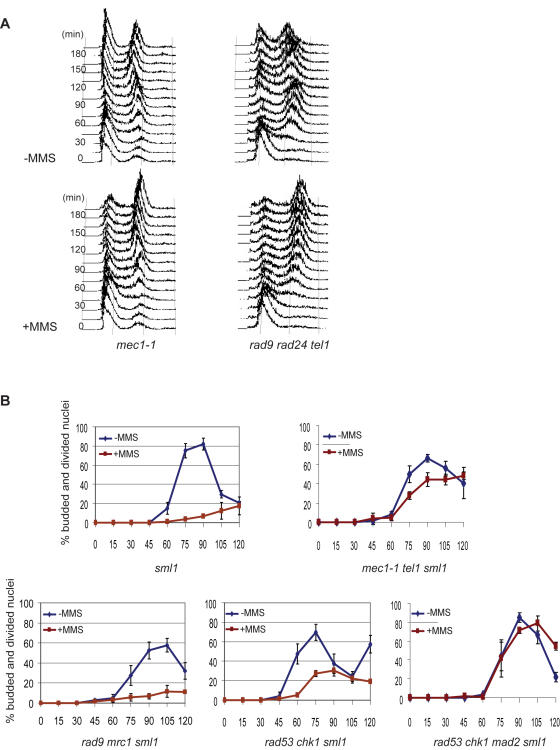
Mec1 and Tel1 activate the SAC in response to MMS. (A) Flow cytometry of *mec1-1* and *rad9 rad24 tel1* cells arrested with α-factor and released to the cell cycle with or without MMS. (B) The percentage of cells that completed anaphase of isogenic *sml1Δ* cells (EKY490) and mutants of the indicated genotypes that were arrested with α-factor and had formed mating projections. The mean numbers of cells are graphed for cells with the indicated genotypes and the standard deviations are represented by error bars. Experiments were repeated independently three times and at least one hundred cells were counted for each time point in each experiment.

The lack of a kinetochore requirement for Mad1, Mad2 and Mad3-dependent APC^Cdc20^ inhibition was surprising because kinetochores are believed to be the source of the signal that activates the SAC [43]–[46]. One possibility for how the SAC proteins respond to DNA damage, independently of the kinetochore, is that they become activated in a DNA damage-dependent manner. We analyzed *mec1* and *tel1* mutants to determine if there was a role of either protein in transducing the signal from DNA damage to the SAC proteins. *MEC1* encodes a PIKK that is homologous to the human ATR and is a central transducer of the checkpoint response in yeast [1],[3]. *TEL1* encodes the related PIKK homologue ATM and plays a lesser role in the DNA damage checkpoint in yeast. *mec1-1* cells, grown in the presence of MMS, arrested with a G2/M content of DNA (Figure 3A). Similarly, *rad9 rad24 tel1* cells delayed with a G2/M content of DNA in response to MMS (Figure 3A) suggesting that the delay is independent of Mec1 and Tel1. We constructed a *mec1 tel1* double mutant to determine if the kinases contributed redundantly in activating the SAC. Only 60% of the *mec1 tel1* cells were viable which precluded analysis by flow cytometry. We used the same assay as described above for *bub3* and *pds1* cells to determine the effect of MMS in *mec1 tel1* cells. Wild type and *mec1* cells arrested prior to anaphase when grown in the presence of MMS but *mec1 mad2* cells completed nuclear division (data not shown). Therefore *mec1* cells, like *rad9 rad24* cells, arrest in mitosis in a Mad2-dependent fashion in response to MMS. Interestingly, *mec1 tel1* cells were unable to arrest and completed nuclear division when grown in the presence of MMS (Figure 3B). Together, these data suggest that Mec1 and Tel1 act redundantly to activate the SAC proteins and inhibit APC^Cdc20^ in response to MMS.

It is possible that the effects of Mec1 and Tel1 on the SAC were indirect. The single mutants lacking either Mec1 or Tel1 may retain sufficient PIKK activity to activate the downstream effector kinases Rad53 and Chk1 and contribute to the pre-anaphase G2/M delay. Perhaps cells lacking both Mec1 and Tel1 do not activate Rad53 and Chk1 and in their absence the SAC is unable to restrain anaphase. This is an important distinction because it would affect the interpretation that the SAC is activated in a Mec1 and Tel1-dependent fashion. The *MEC1* gene is essential and *mec1-1* cells are viable in the presence of a second mutation, *sml1*, that suppresses the *mec1-1* lethality but does not suppress the DNA damage checkpoint phenotype. We used the same assay as described above for *bub3, pds1* and *mec1 tel1* cells to determine if there was a an effect of MMS on mitotic progression in a set of isogenic strains lacking Sml1 and proteins of the DNA damage checkpoint and the SAC. The *sml1* cells, treated with MMS, behaved like wild type cells (Figure 1A) and arrested in mitosis prior to anaphase in contrast to the *mec1 tel1 sml1* cells described above (Figure 3B). *rad9 mrc1 sml1* cells that lack the S-phase checkpoint delayed prior to anaphase when grown in the presence of MMS (Figure 3B). *rad53 chk1sml1* cells also delayed prior to anaphase when grown in the presence of MMS although a small percentage of cells entered into anaphase. However, the delay in *rad53 chk1sml1* cells was abrogated by deleting *MAD2* (*rad53 chk1 mad2 sml1*) as shown in Figure 3B. Therefore a partially activated DNA damage checkpoint is not sufficient to explain the entire pre-anaphase delay in MMS treated *rad9 rad24* cells. We conclude that the SAC is sufficient to delay anaphase in the absence of the DNA damage checkpoint and that the SAC is activated in a Mec1 and Tel1 dependent fashion. An important study has recently shown that there is PIKK-dependent phosphorylation of SAC proteins in response to DNA damage in human cells suggesting that SAC proteins are substrates of ATM and ATR in response to DNA damage [47]. Together the data suggest that there may be an evolutionarily conserved response of cells to DNA damage that involves ATM and ATR-dependent phosphorylation of SAC proteins that helps to enforce a mitotic arrest in response to DNA damage.

**Figure 4:**
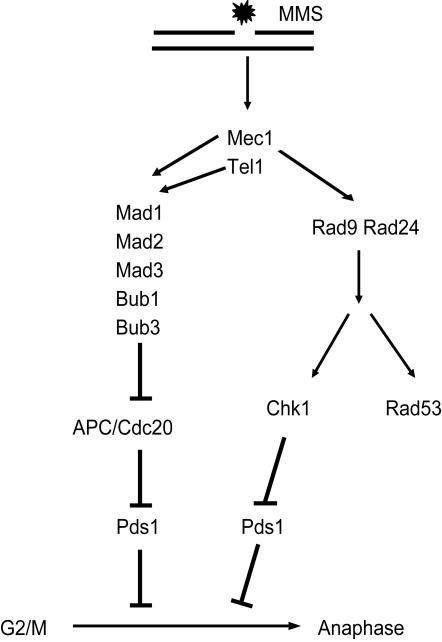
A model for the role of the SAC in response to DNA damage. The lesion (indicated by the star) activates Mec1 and Tel1 which inhibits anaphase by phosphorylating Pds1 through the DNA damage checkpoint and independently inhibits Pds1 turnover by inhibiting APC^Cdc20^ through the activity of the SAC.

Our data extends the previous observation that the SAC mediates a mitotic delay in response to multiple lesions affecting DNA replication [22]–[25],[48]. Two previous studies have shown that the SAC contributes to survival of cells lacking the DNA damage checkpoint when cells are treated with MMS or when compromised for DNA replication [24],[25]. Our data extend these previous studies in two important ways. We have shown that the SAC inhibits APC^Cdc20^ when cells are grown in the presence of MMS and SAC-dependent inhibition does not require a functional kinetochore. In addition, we have shown that the SAC depends on the PIKKs Mec1 and Tel1. Our data are summarized in a model in Figure 4. Tel1 and Mec1, in response to MMS (and other mutations and treatments), activate both the DNA damage checkpoint and the SAC. The DNA damage checkpoint and the SAC converge on Pds1, by independent mechanisms, to restrain anaphase. One possible reason is that the DNA damage checkpoint recruits the SAC as a backup to assure that cells do not enter anaphase. MMS treatment causes stalled replication forks [49]. Cells will activate the DNA damage checkpoint only after they surpass a threshold of stalled replication forks, presumably because stalled and active forks are similar in structure [50],[51]. This threshold assures that the DNA damage checkpoint does not interfere with normal replication. A cell that enters into mitosis with stalled replication forks, below the threshold, could initiate a catastrophic mitosis. If cells arrest because of some threshold of stalled replication forks, then this would constitute a new checkpoint for the completion of DNA replication. Such a checkpoint is controversial [52] but the exciting possibility that Mec1 and Tel1 activates the SAC to achieve a cell cycle arrest warrants further investigation.

## Materials and Methods

### Yeast Strains and Plasmid Construction

All strains were derivatives of W303 (*MAT* **a** or *MAT*α *ade2-1 trp1-1 can1-100 leu2-3,112 his3-11,15 ura3-1*) and are listed in Table S1. Cells were arrested using the mating pheromone, α-factor at 5 µM for *BAR1* strains and 0.1 µM for *bar1* strains. Cells were released from α-factor by washing in water for three times and released into fresh pre-warmed medium. The temperature sensitive strain, *rad9 rad24 ndc10-1*, was grown at 23°C (permissive) and tested at 35°C (restrictive).

Standard yeast genetics techniques and media were used [53]. Cells were grown in YPD medium (1% yeast extract, 2% Bacto Peptone, 2% Glucose, 40 mg of adenine per liter). Yeast transformations were by the lithium acetate method [54]. Epitope-tagged alleles *PDS1-13MYC-HIS* were constructed by PCR-mediated one-step gene replacements [55].

The *ndc10-1* mutant was obtained as by double fusion PCR [53]. Deletion of *MAD1*, *MAD3*, *BUB1*, and *BUB3* genes were generated by PCR and transformation for each coding region was replaced by the KanMx4 or ClonNAT (NAT) genes by one-step gene replacement. The *CDC20-127* dominant allele was made from PCR and transformed to wild type and *rad9 rad24* strains [40]. Other mutants were made by standard tetrad genetics.

### Yeast Cell Cycle Experiments

Cells were grown to O.D. of 2.0 overnight in YPD medium. For synchrony, cells were diluted to O.D. of 0.2 in YPD medium for *bar1* deletion strains or acidic YPD (pH 3.5) medium (*BAR1* strains) with α-factor. Cells were monitored under microscope to arrest 85–100% as unbudded cells typically after 2.5–3 hours. Cells were washed with water and resuspended in fresh medium under experimental conditions. Methylmethane sulfonate (MMS, Sigma M-4016) concentration was 0.01% V/V. For experiments with the temperature sensitive strain *rad9 rad24 ndc10-1*, wild type and mutant cells were grown and arrested with α-factor at 23°C. They were shifted to 35°C for 1 hour to inactivate Ndc10 and then released in fresh medium at 35°C with or without MMS. At each time point and for each strain, cells were taken for DAPI staining or FACScan (flow cytometry) using Sytox Green (Molecular Probes, Inc.) and western blot analysis. Western blots were with mouse monoclonal anti-Myc antibody 9E10, or rabbit anti-Tub2 antibody FY124, a generous gift from Frank Solomon (MIT), for tubulin loading controls. Flow cytometry was as previously described [56] and performed at the University of Virginia core fluorescence-activated cell sorting facility. Every strain was tested independently at least twice and up to six times by flow cytometry. Nuclear division for cells stained with Sytox green or DAPI was determined using a Nikon E600 microscope equipped with epifluorescence. At least 100 cells were counted for each time point.

### CHEF (Clamped Homogeneous Electric Fields)

Cells were arrested with α-factor and after 3 hrs at 23°C they were washed and released in fresh media with or without 0.01% MMS. The cells arrested in S phase were treated with 0.1 M Hydroxyurea (HU, Sigma H-862). Samples were taken in every hour. Plugs for CHEF gels were prepared as soon as the cells were sampled according to manufacturer’s instructions (BioRad). Samples were subjected to CHEF; 120° field angle, 6 V/cm, initial switch time of 60 s, final switch time of 120 s for 21 h at 11°C.

## Supporting Information

Figure S1 Cellular morphology of wild type and *mad2* cells. Wild type (WT) and mutant cells with the indicated genotypes that were untreated (−MMS) and treated (+MMS) by growth in YPD medium with or without 0.01% MMS. Cells were arrested with α-factor, released and assayed every fifteen minutes. The graphs show the percentages of G2/M cells determined from the FACScan profiles. Solid lines were mean values of two (marked without line in *rad9 rad24* and *mad2*) or at least three independent experiments (in WT and *rad9 rad24 mad2*). Flow cytometry figures are duplicates from independent experiments. Upper panel is without MMS and lower panel is with MMS. (A) WT, (B) *rad9 rad24*, (C) *mad2*, (D) *rad9 rad24 mad2*.
(4.70 MB EPS)

Figure S2 The percentage of cellular morphology from Figure S1. Budding was determined by phase contract microcopy and nuclear division was assayed using Sytox green staining and detected by epi-fluorescence microscopy.
(1.16 MB EPS)

Figure S3 Cellular morphology of *rad9 rad24 mad1*, *rad9 rad24 mad3*, *rad9 rad24 bub1*, and *rad9 rad24 ndc10-1* cells. Flow cytometry of mutant cells with the indicated genotypes that were untreated (−MMS) and treated (+MMS) by growth in YPD medium with or without 0.01% MMS. The graphs showed the percentages of G2/M peak determined from the FACScan profiles. Solid lines were mean values of two (*rad9 rad24 mad1*, *rad9 rad24 mad3*, and *rad9 rad24 ndc10-1*) or three experiments (*rad9 rad24 bub1*). Flow cytometry is from duplicate experiments. Upper panel is without MMS and lower panel is with MMS. (A) *rad9 rad24 mad1*, (B) *rad9 rad24 mad3*, (C) *rad9 rad24 bub1*, (D) *rad9 rad24 ndc10-1*.
(5.85 MB EPS)

Figure S4 The percentage of cellular morphology from Figure S3. Budding was determined by phase contract microcopy and nuclear division was assayed using Sytox green staining and detected by epi-fluorescence microscopy.
(1.18 MB EPS)

Figure S5 Cellular morphology *CDC20-127* and *CDC20-127* *rad9 rad24 cells*. Flow cytometry of wild type cells and mutant cells with the indicated genotypes that were untreated (−MMS) and treated (+MMS) by growth in YPD medium in the presence or absence of 0.01% MMS. The graphs showed the percentages of G2/M peak as determined by FACScan profiles. Solid lines are mean values of repeated experiments. Flow cytometry figures from duplicate, independent experiments. Upper panel is without MMS and lower panel is with MMS. (A) *CDC20-127*, (B) *CDC20-127 rad9 rad24*. (C) Pds1-13 Myc stability of *CDC20-127* and *CDC20-127 rad9 rad24* cells. Endogenous Pds1 was tagged with 13 copies of the Myc epitope. Upper half was in the absence of MMS and lower half was in the presence of MMS. Western blots with anti-Tub2 (*β*-tubulin) were for loading control. (D) Budding was determined by phase contract microcopy and nuclear division was assayed using Sytox green staining and detected by epi-fluorescence microscopy.
(9.22 MB EPS)

Table S1 *Saccharomyces cerevisiae* strains used in this study.
(0.05 MB DOC)
