# Supplementary material for: DNA Damage Activates the SAC in an ATM/ATR-Dependent Manner, Independently of the Kinetochore
Source: PLoS Genet. 2008 Feb 29;4(2):e1000015. doi: 10.1371/journal.pgen.1000015 (PMC2265443; doi:10.1371/journal.pgen.1000015)
Supplement: Table S1 — Saccharomyces cerevisiae strains used in this study. (0.05 MB DOC) [file pgen.1000015.s006.doc]

**Table S1.** *Saccharomyces cerevisiae* strains used in this study.*

| Strains | Genotype | |
| --- | --- | --- |
| W303 | *MAT*α *bar1::hisG* | Ted Weinert |
| EKY6211 | *MAT***a** *bar1::hisG PDS-13Myc:HIS3* | This study |
| EKY6254 | *MAT***a** *bar1::hisG mad2::KanMX4 PDS1-13Myc:HIS3* | This study |
| EKY9613 | *MAT***a** *rad9::HIS3 rad24::TRP1 PDS1-13Myc:HIS3* | This study |
| EKY9619 | *MAT***a** *rad9::HIS3 rad24::TRP1 PDS1-13Myc:HIS3 mad2::URA3* | This study |
| EKY6213 | *MAT***a** *bar1::hisG ndc10-1:URA3 PDS-13Myc:HIS3* | This study |
| EKY9617 | *MAT***a** *rad9::HIS3 rad24::TRP1 ncd10-1:URA3 PDS1-13Myc:HIS3* | This study |
| EKY9622 | *MAT***a** *rad9::HIS3 rad24::TRP1 PDS1-13Myc:HIS3 mad1::KanMX4* | This study |
| EKY9623 | *MAT***a** *rad9::HIS3 rad24::TRP1 PDS1-13Myc:HIS3 mad3::KanMX4* | This study |
| EKY9620 | *MAT***a** *rad9::HIS3 rad24::TRP1 PDS1-13Myc:HIS3 bub1::KanMX4* | This study |
| EKY9624 | *MAT***a** *rad9::HIS3 rad24::TRP1 PDS1-13Myc:HIS3 bub3::NAT* | This study |
| EKY6220 | *MAT***a** *bar1::hisG CDC20-127:KlURA3 PDS-13Myc:HIS3* | This study |
| EKY6222 | *MAT***a** *rad9::HIS3 rad24::TRP1 CDC20-127 PDS1-13Myc:HIS3* | This study |
| EKY9910 | *MAT***a** *rad9::HIS3 rad24::TRP1 PDS1-13Myc:HIS3 ade2-1 tel1::LEU2* | This study |
| EKY9515 | *MAT***a** *bar1 pds1::LEU2 rad9::HIS3 rad24::TRP1* | This study |
| DLY258 | *MAT***a** *bar1::hisG mec1-1 sml1* | Ted Weinert |
| EKY2520 | *MAT***a** *bar1::hisG mec1-1 sml1 ura3 leu2 his3 trp1 ade2 rad5 mad2::KanMX* | This study |
| EKY2521 | *MAT***a** *bar1::hisG mec1-1 sml1 tel1::URA3* | This study |
| EKY490 | *MAT****a*** *sml1Δ::KANMX4* | This study |
| EKY499 | *MAT****a*** *rad9::Sphis5+ mrc1Δ::HIS3 sml1Δ::KANMX4* | This study |
| DMP 4359/4C | *MAT****a*** *rad53Δ::HIS3 chk1Δ::HIS3 sml1Δ::KANMX4* | Maria  Longhese |
| DMP 4359/9A | *MAT***a** *rad53::HIS3 chk1Δ::HIS3 sml1Δ::kanMX4 mad2Δ::HIS3* | Maria  Longhese |

* All strains are isogenic with W303 and are *ade2-1 trp1-1 can1-100 leu2-3,112 his3-11,15 ura3-1 GAL+ psi+ ssd1-d2* and have the indicated mutations.
